# Application of Permutation Entropy and Permutation Min-Entropy in Multiple Emotional States Analysis of RRI Time Series

**DOI:** 10.3390/e20030148

**Published:** 2018-02-26

**Authors:** Yirong Xia, Licai Yang, Luciano Zunino, Hongyu Shi, Yuan Zhuang, Chengyu Liu

**Affiliations:** 1 School of Control Science and Engineering, Shandong University, Jinan, 250061, China; 2 Centro de Investigaciones Ópticas (CONICET La Plata—CIC), C.C. 3, 1897 Gonnet, Argentina; 3 Departamento de Ciencias Básicas, Facultad de Ingeniería, Universidad Nacional de La Plata (UNLP), 1900 La Plata, Argentina; 4 School of Instrument Science and Engineering, Southeast University, Nanjing 210018, China

**Keywords:** permutation entropy, permutation min-entropy, emotion recognition, heart rate variability, electrocardiogram (ECG), 87.85.Ng, 05.45.Tp, 87.19.Hh, 87.19.ug, 87.19.uj

## Abstract

This study’s aim was to apply permutation entropy (PE) and permutation min-entropy (PME) over an RR interval time series to quantify the changes in cardiac activity among multiple emotional states. Electrocardiogram (ECG) signals were recorded under six emotional states (neutral, happiness, sadness, anger, fear, and disgust) in 60 healthy subjects at a rate of 1000 Hz. For each emotional state, ECGs were recorded for 5 min and the RR interval time series was extracted from these ECGs. The obtained results confirm that PE and PME increase significantly during the emotional states of happiness, sadness, anger, and disgust. Both symbolic quantifiers also increase but not in a significant way for the emotional state of fear. Moreover, it is found that PME is more sensitive than PE for discriminating non-neutral from neutral emotional states.

## 1. Introduction

Emotion recognition by using physiological signals, including electroencephalogram (EEG) [1], photoplethysmography (PPG) [2], skin temperature [3], and electrocardiogram (ECG) [4], has attracted increasing attention. Among them, ECG signals and their related RR interval (RRI) time series are mostly used.

Time-domain and frequency-domain indices were conventionally used for emotional state recognition. Guo et al. [5] applied time-domain indices, mean, coefficient of variation of RR intervals, standard deviation of the RR intervals, and standard deviation of the successive differences of the RR intervals together with a support vector machine classifier, reaching 50.3% of correct rate to discriminate negative and positive emotional states. With frequency-domain indices—low-frequency power (LF), high-frequency power (HF) and low-frequency power to high-frequency power ratio (LF/HF)—54.3% of correct rate has been obtained. Kim et al. [6] used spectrum, amplitude, mean, maximum, and standard deviation together with a support vector machine classifier to achieve an accuracy of 61.8% for classifying the emotional states of sadness, stress, surprise, and anger. Jang et al. [7] used mean, HF, and LF of RRI time series and other physiological signals to classify boredom, pain, and surprise and reached 84.7%, the best classification rate. Ma et al. [8] used mean, median, standard deviation, maxima, minima, maximal interval, and spectrum average of heart rate variability (HRV) and obtained an average of 88.88% recognition rate for classifying joy and sadness.

The use of nonlinear dynamic methods can enhance the accuracy of emotion recognition. Indeed, Valenza et al. [9] evaluated deterministic chaos, recurrence plots, and detrended fluctuation analysis from HRV, respiration activity, and electrodermal response series and attained an accuracy of 90%. Features from the instantaneous spectrum and bispectrum, as well as the dominant Lyapunov exponent, were extracted from RRI time series and an overall accuracy of 79.29% was achieved in recognizing four emotional states (happiness, sadness, anger, and relaxation) with 79.15% on the valence axis and 83.55% on the arousal axis [10]. Goshvarpour et al. [11] used Poincare plot indices, an efficient nonlinear tool to extract features from RRI time series, and obtained the best classification rate of 97.45% to distinguish the neutral, happiness, relaxation, sadness, and fear emotional states.

Among the nonlinear approaches, permutation entropy (PE) plays an important role because of its robust, fast, and easy computation. This entropic measure, introduced by Bandt and Pompe [12], is based on the comparison between the neighboring values of a time series. PE quantifies the diversity of the ordinal pattern distribution. It is maximal in the case of a uniform distribution (white noise). When some ordinal patterns appear more frequently than others, then the PE decreases. It has been applied in different fields [13,14,15,16]. Within the physiological field, PE has been frequently used to analyze EEG signals for different purposes [17,18,19]. PE has also been used to characterize the RRI time series. Actually, PE of HRV was confirmed to separate workload levels between different stages of anesthesia [20]. Aziz and Arif [21] found that the PE of the RRI time series of normal sinus rhythm subjects becomes greater than that of the RRI time series from congestive heart failure subjects. Graff et al. [22] reported that PE is significantly different between groups with cardio depressive or negative results for the head-up tilt test.

However, little attention has been paid so far to PE of the RRI time series for emotion recognition. To the best of our knowledge, the research developed by García-Martínez et al. [23] is the only one in which this symbolic entropic measure has been considered for automatic recognition of emotions. More precisely, EEG records have been analyzed for discerning between emotions of calm and stress. Results obtained confirm that PE is useful for this purpose. In addition, permutation min-entropy (PME) is an improved symbolic alternative for identifying the existence of hidden temporal correlations in time series and it allows a better discrimination of time series with similar temporal correlations [24]. It is defined as the Rényi PE when the order *q* goes to infinity. It can be shown that, in this limit, the min-entropy is a function of the highest probability only. Since it is estimated by implementing the Bandt and Pompe scheme, all the advantages associated with this symbolic method are naturally inherited [24]. PME improves PE in this sense. It has not been previously implemented for characterizing different emotional states. Thus, this study aimed to analyze how the PE and PME of the RRI time series change during the happiness, sadness, anger, fear, and disgust emotional states when comparing with the neutral emotional state. The effect of using different embedding delays to estimate PE and PME has also been considered in this research.

## 2. Methods

### 2.1. Subjects

Sixty subjects with a mean age of 23 years, a height of 169 centimeters and a weight of 61 kilograms were enrolled in this study (30 females and 30 males). All subjects were healthy graduate students with no history of psychotropic medication or abuse of alcohol, confirmed by a medical examination at the University Hospital. All subjects signed the consent forms in the study. Before and after the signal recording, heart rate (HR), systolic blood pressure (SBP), and diastolic blood pressure (DBP) were measured using the OMRON HEM-7051 electronic sphygmomanometer device. The details of the subjects are summarized in Table 1.

### 2.2. Experimental Procedure and Data Collection

**Figure 1:**
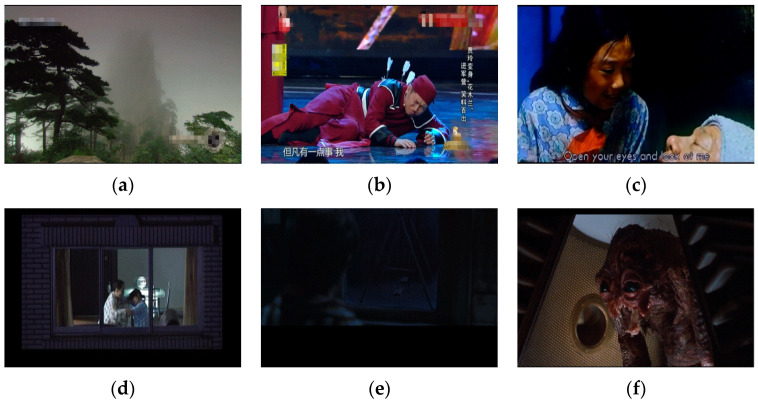
Still images from emotion-stimulating videos for inducing (**a**) neutral, (**b**) happiness, (**c**) sadness, (**d**) anger, (**e**) fear, and (**f**) disgust emotions.

ECG signals were collected under neutral and five non-neutral emotional states of happiness, sadness, anger, fear, and disgust. Video clips (each about ten minutes) of ‘World Heritage In China’ (a documentary), ‘Top Funny Comedian’ (a situation comedy), ‘Nuan Chun’ (a touching movie), ‘Never Talk to Strangers’ (a domestic violence television series), ‘The Conjuring’ (a horror movie), and ‘The Fly’ were used to induce the emotional states of neutral, happiness, sadness, anger, fear, and disgust, respectively. Figure 1 shows still images from the six emotion-stimulating video clips.

Subjects were tested in a quiet room and while sitting in a comfortable chair. Ten minutes were given to the subjects to calm themselves. Before playing the emotion-stimulating videos, subjects with ECG electrodes watched a 1-minute video to adapt to the experimental conditions. Then, emotion-stimulating videos were played in randomized order to the subjects. A gap of 10 min between videos was used to make sure the subjects could recover from the previous emotional state. ECG signals were recorded by a RM6240B signal acquirement system with standard leads at a rate of 1000 Hz. For each emotional state, ECG signals were acquired from the fourth to eighth minute (for 5 min) of each video clip.

After signal collection, a sym8 wavelet filter was used to remove the high-frequency interference and baseline drift in the ECG signal [25]. R peaks were detected by the difference threshold algorithm [26]. Additionally, we checked each ECG signal and manually removed the error R peaks before generating the RRI time series. The RRI time series has a length of about 300 to 400 intervals for each emotional state of each subject.

### 2.3. Permutation Entropy and Permutation Min-Entropy

A time series {*x*(*t*), *t* = 1, 2, …, *N* } can be partitioned into subsets of length *D* and lag *τ as* {*X*(*t*) = [*x*(*t*), *x*(*t* + *τ*),…, *x*(*t* + (*D* − 1)*τ*)], *t* = 1, 2,…, *N* − (*D* − 1)*τ*)}, where *D* is the embedding dimension and τ is the embedding delay. *X*(*t*) is rearranged in an ascending order and an ordinal pattern *π* is created. For *D* different numbers, there will be *D*! possible ordinal patterns. For example, when *D* = 3, there are 3! = 6 ordinal patterns. They are denoted as (supposing *τ* = 1):

(1) $$\begin{array}{cc} x(t)<x(t+1)<x(t+2) & \left\{123\right\}\\ x(t)<x(t+2)<x(t+1) & \left\{132\right\}\\ x(t+1)<x(t)<x(t+2) & \left\{213\right\}\\ x(t+1)<x(t+2)<x(t) & \left\{231\right\}\\ x(t+2)<x(t)<x(t+1) & \left\{312\right\}\\ x(t+2)<x(t+1)<x(t) & \left\{321\right\} \end{array}$$

The above definitions apply to time series that have a continuous distribution so that equal values are very rare. Otherwise, equalities may cause a deviation from true PE [27]. In order to avoid this drawback, a very small amount of noise can be added to break ties.

The relative frequency of each *π* (# means number) is defined as:

(2) $$p(\pi )=\frac{\#\{X(t)\;\mathrm{has}\;\mathrm{type}\;\pi \;\}}{N-(D-1)\tau }$$

The permutation entropy is defined as:

(3) PE=−∑p(π)ln p(π)

It is clear that the bound of *PE* is [0, *lnD*!]. It is also worth remarking here that the condition *N* >> *D*! should be satisfied in order to achieve a reliable estimation of the relative frequency for the different ordinal patterns. As an illustrative example, an artificial time series *x*(*t*) ={3, 5, 2, 1, 4, 8, 5, 6} can be symbolized with ordinal patterns as follows:

(4) $$\begin{array}{cc} \left[3\;5\;2\right] & \left\{312\right\}\\ \left[5\;2\;1\right] & \left\{321\right\}\\ \left[2\;1\;4\right] & \left\{213\right\}\\ \left[1\;4\;8\right] & \left\{123\right\}\\ \left[4\;8\;5\right] & \left\{132\right\}\\ \left[8\;5\;6\right] & \left\{231\right\} \end{array}$$

The PE of this toy time series is calculated as:

(5) $$\begin{array}{cc} \mathrm{PE} & =-1/6\ln(1/6)-1/6\ln(1/6)-1/6\ln(1/6)-1/6\ln(1/6)-1/6\ln(1/6)-1/6\ln(1/6)\\ & \approx 1.7918 \end{array}$$

For the purpose of identifying the presence of hidden correlational structures in complex time series, Zunino et al. [24] defined a new entropy, the permutation min-entropy (PME). It is applicable to noisy real-time series from all classes of systems, deterministic and stochastic, without the need to require any assumption about the generating process. PME is defined as Equation (6). It is obvious that the bound of PME is [0, *lnD*!]:

(6) $$\mathrm{PME}=-\ln\left(\begin{array}{c} \max\\ i=1,\ldots ,D! \end{array}p(\pi_{i})\right)$$

Taking into account the length of the RRI time series (300–400 data points), the embedding dimensions *D* = 3 and *D* = 4 and embedding delays *τ* between 1 and 10 have been considered in this paper.

### 2.4. Statistical Analysis

In this study, PE and PME were compared as discriminators of different emotional states. Normal distributions of the results were tested by the Kolmogorov–Smirnov test. Throughout all results, data were normally distributed. Hence, the differences between the neutral and the five non-neutral emotional states (happiness, sadness, anger, fear, disgust) were compared by paired Student’s *t*-test. All statistical analyses were performed using the SPSS software (Version 20, IBM, USA). The statistical significance was accepted at *P* < 0.05.

## 3. Results

### 3.1. Permutation and Permutation Min-Entropy Analysis

**Figure 2:**
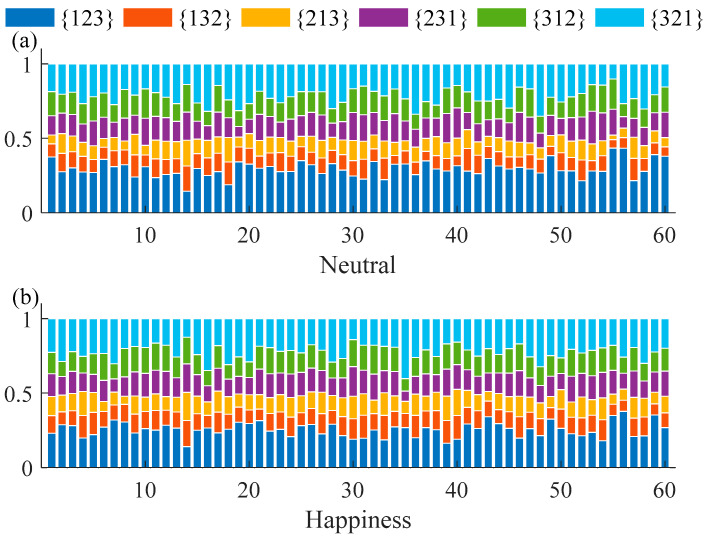
Distributions of ordinal patterns with *D* = 3 and *τ* = 1 of the neutral and happiness emotional states.

The value of permutation entropy is based on the distribution of ordinal patterns. The distribution of ordinal patterns during the six emotional states was observed. The distribution of ordinal patterns with *D* = 3 and *τ* = 1 of the neutral and happiness emotional states of 60 subjects are shown in Figure 2. Similar results were obtained for the other emotional states. We found that patterns {123} and {321}, {132} and {213}, {231} and {312}, respectively, had similar frequency percentages as Graff et al.’s study [28]. Subtle changes in the distribution of the ordinal patterns could reflect on the value of PE, and also on PME.

**Figure 3:**
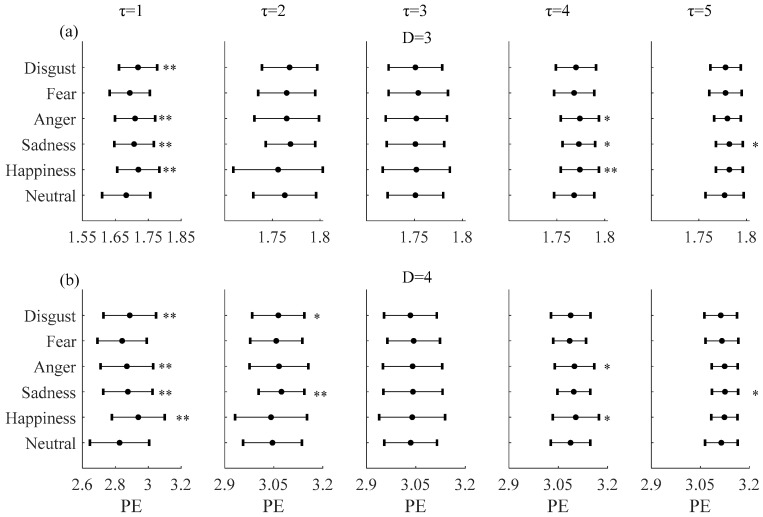
Mean and SD of permutation entropy (PE) with (**a**) *D* = 3, (**b**) *D* = 4 and embedding delays *τ* between 1 and 5 of the RRI time series during six emotional states. ** mean *P* < 0.01. * mean *P* < 0.05.

**Figure 4:**
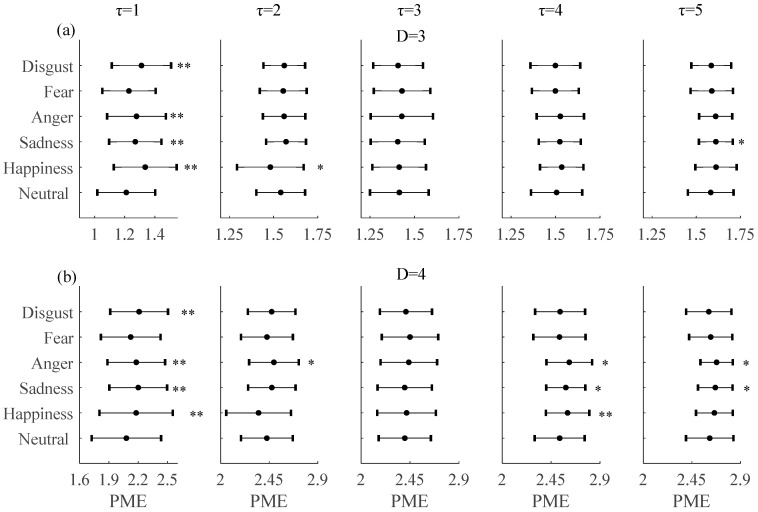
Mean and SD of permutation min-entropy (PME) with (**a**) *D* = 3, (**b**) *D* = 4 and embedding delays *τ* between 1 and 5 of the RRI time series during six emotional states ** mean *P* < 0.01. * mean *P* < 0.05.

The effect of embedding delays on PE and PME was analyzed and it was found that PE and PME were near to theoretical maximum when *τ* was higher than 5. Furthermore, the difference between neutral and non-neutral emotional states with *τ* ∈ [6, 10] was similar to *τ* = 1. Mean and SD of PE and PME with embedding dimension *D* = 3 and *D* = 4 for *τ* ∈ [1, 5] are plotted in Figure 3 and Figure 4, respectively. The results show that there are significant differences in PE and PME when *τ* = 1 for four emotional states (happiness, sadness, anger, and disgust).

Hereafter, taking into account these findings, *τ* = 1 was chosen as the optimal one for distinguishing between neutral and non-neutral emotional states with embedding dimensions *D* = 3 and *D* = 4.

### 3.2. Comparisons between Neutral and Non-Neutral Emotional States

**Figure 5:**
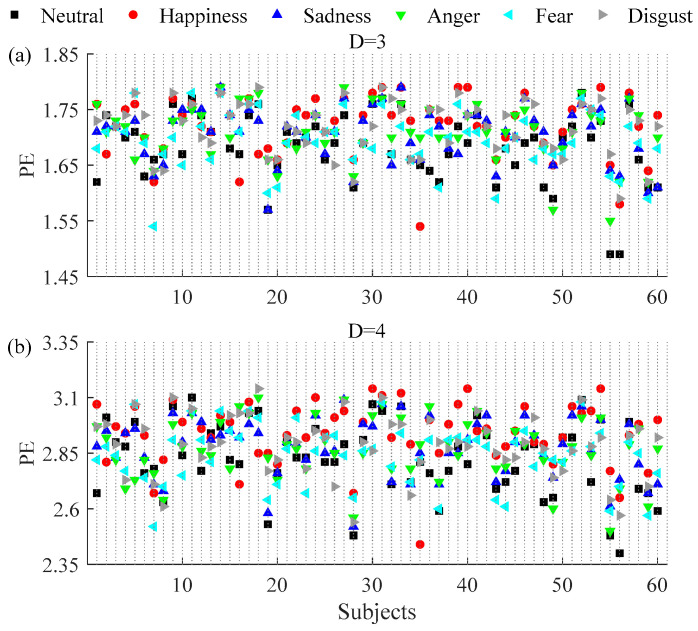
PE results with (**a**) *D* = 3, *τ* = 1 and (**b**) *D* = 4, *τ* = 1 for each of the 60 subjects under the six emotional states.

Figure 5 shows the values estimated for the PE (with (a) *D* = 3 and *τ* = 1 and (b) *D* = 4 and *τ* = 1) for each of the 60 subjects under the six emotional states. It was found that PE of the non-neutral emotions for the mass of subjects was higher than the neutral emotional state. The statistical results (mean and SD) are shown in the first plots of Figure 3a,b, respectively. Compared with the neutral emotional state (1.6834 ± 0.06), PE increased with embedding dimension *D* = 3 in all five non-neutral emotional states: happiness (1.7205 ± 0.05, *P* < 0.01), sadness (1.7065 ± 0.05, *P* < 0.01), anger (1.7098 ± 0.04, *P* < 0.01), fear (1.6937 ± 0.05, *P* = 0.10), and disgust (1.7186 ± 0.05, *P* < 0.01). With embedding dimension *D* = 4 and comparing with the neutral emotional state (2.8295 ± 0.16), PE also increased in all five non-neutral emotional states: happiness (2.9395 ± 0.14, *P* < 0.01), sadness (2.8762 ± 0.13, *P* < 0.01), anger (2.8701 ± 0.14, *P* < 0.01), fear (2.8413 ± 0.13, *P* = 0.32), and disgust (2.8876 ± 0.14, *P* < 0.01). The increases are significant for all except the fear emotional state.

**Figure 6:**
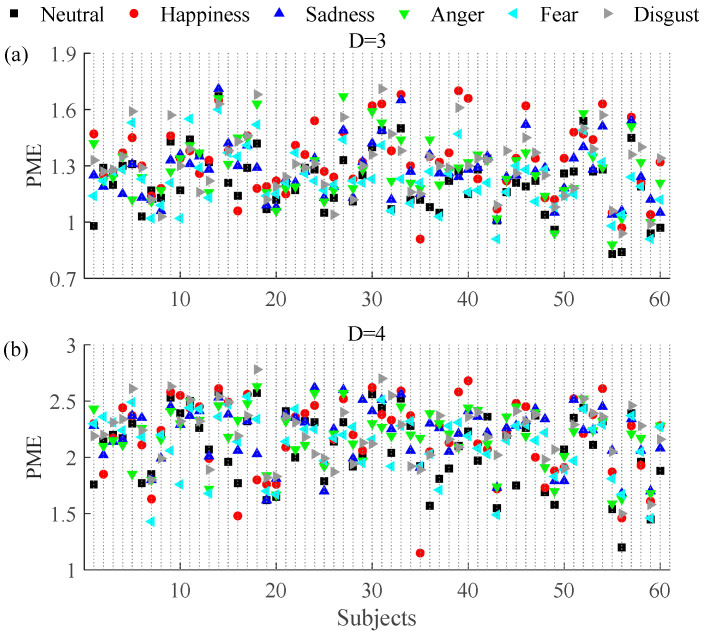
PME results with (**a**) *D* = 3, *τ* = 1 and (**b**) *D* = 4, *τ* = 1 for each of the 60 subjects under the six emotional states.

Figure 6 shows the values estimated for the PME (with (a) *D* = 3 and *τ* = 1 and (b) *D* = 4 and *τ* = 1) for each of the 60 subjects under the six emotional states; the results are similar to PE. The first plots of Figure 4a,b show the results obtained for the PME (also with (a) *D* = 3 and *τ* = 1 and (b) *D* = 4 and *τ* = 1). Compared with the neutral emotional state (1.2099 ± 0.14), PME increased with embedding dimension *D* = 3 in all five non-neutral emotional states: happiness (1.3360 ± 0.14, *P* < 0.01), sadness (1.2699 ± 0.12, *P* < 0.01), anger (1.2781 ± 0.14, *P* < 0.01), fear (1.2282 ± 0.13, *P* = 0.27), and disgust (1.3114 ± 0.14, *P* < 0.01). With embedding dimension *D* = 4 and compared with the neutral emotional state (2.0870 ± 0.32), PME also increased in all five non-neutral emotional states: happiness (2.1778 ± 0.34, *P* < 0.01), sadness (2.1993 ± 0.26, *P* < 0.01), anger (2.1790 ± 0.26, *P* < 0.01), fear (2.1222 ± 0.27, *P* = 0.22), and disgust (2.2083 ± 0.26, *P* < 0.01). Similar to the PE, the change in PME is significant for all except the fear emotional state.

**Figure 7:**
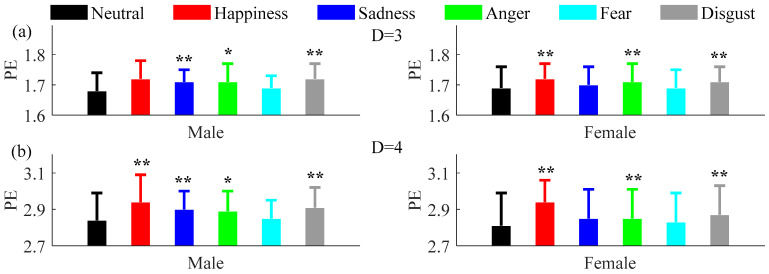
Mean and SD of PE and PME for males and females with (**a**) *D* = 3, *τ* = 1 and (**b**) *D* = 4, *τ* = 1 under the six emotional states. ** mean *P* < 0.01.* mean *P* < 0.05.

**Figure 8:**
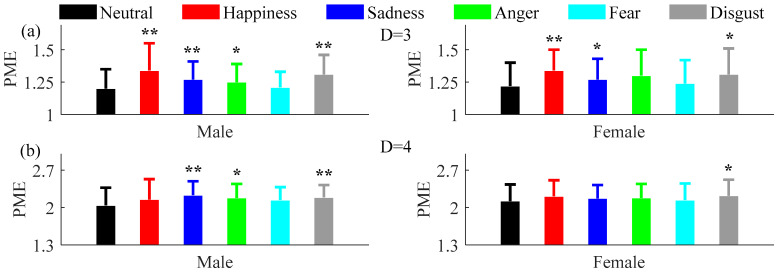
Mean and SD of PME for males and females with (**a**) *D* = 3, *τ* = 1 and (**b**) *D* = 4, *τ* = 1 under the six emotional states. ** mean *P* < 0.01.* mean *P* < 0.05.

Since the number of females and males enrolled in this research were the same, possible differences due to gender were tested. As shown in Figure 7 and Figure 8, the results show that compared with the neutral emotional state, both PE and PME increased in all five non-neutral emotional states when embedding dimension *D* = 3 and *D* = 4.

### 3.3. Comparisons of Permutation and Permutation Min-Entropy

**Figure 9:**
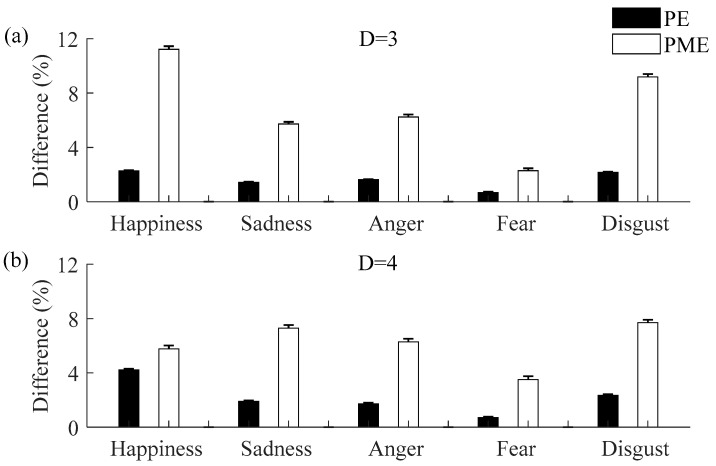
Relative increments of PE and PME with (**a**) *D* = 3 and (**b**) *D* = 4 during the non-neutral emotional states when comparing with the neutral emotional state (mean and standard error).

To analyze which of the two symbolic entropies is more sensitive for discriminating emotional states, Figure 9 shows the relative increment associated with the PE and PME for the non-neutral emotional states compared to the neutral emotion state with both embedding dimensions (*D* = 3 and *D* = 4). We observed that the relative increments of the PME are larger than those obtained for the PE for all non-neutral emotional states for the two values of *D*. Thus, PME seems to be more sensitive than PE for characterizing emotional states.

## 4. Discussion

PE and PME are quantifiers of nonlinear dynamic characteristics that could evaluate the inherent complexities of the RRI time series under different emotional states. In this study, we found that PE and PME increase among five non-neutral emotional states, indicating the higher randomness of their associated RRI time series. Moreover, PME appears to be more sensitive for discriminating non-neutral from neutral emotional states. Previous studies showing that other nonlinear measures change during non-neutral emotional states when comparing with neutral emotional states agree with our results that emotions affect cardiac activity [29,30].

Cardiac activity is under the control of the autonomic nervous system [31]. A previous study reported a significant reduction in the entropy related to the RRI time series during head-up tilting, and the results indicate that a sympathetic dominance increases regularity leading to a complexity reduction [32]. On the other hand, Porta et al. [33] found that a high dose of atropine, blocking the vagal system, significantly reduces the RRI time series complexity compared to baseline conditions, while sympathetic blocking with propranolol does not have any effect on the RR interval series complexity. Thus, the authors concluded that vagal efferent activity is the main contributor to the RRI time series complexity and complexity decreases due to a vagal dominance reduction. However, Weippert et al. [34] found that a reduction in complexity after dual autonomic blocking is not as strong as after vagal blocking alone. Therefore, they indicated that both vagal and sympathetic modulations contribute to RRI time series complexity. From all the above-mentioned studies, we hypothesize that subjects under happiness, sadness, fear, anger, and disgust emotional states have increased vagal activity and decreased sympathetic modulation, making the RRI time series more random.

This study has some limitations that should be considered in the future. First, all of the subjects enrolled were healthy young people. To further investigate this concern, a large number of subjects and patients with different emotional diseases will be studied. Second, the experiment lasted about an hour and a half for each subject. Long experiments may cause subjects to feel tired, which may affect emotion stimulation. Third, the length of the time series is short (around 300/400 values) and discrimination between neutral and non-neutral emotional states is only on average. A better discrimination may be reached with longer records.

In conclusion, this study is the first study to compare PE and PME from RRI time series during different emotional states. PE and PME have been found to have a significant difference for non-neutral emotional states when compared with neutral emotional states. This study could help to better understand the differences between the RRI time series of different emotional states.

## Figures and Tables

**Table 1:** Basic clinical characteristics from all 60 subjects.

Variables	Male	Female	Total
Number of subjects	30	30	60
Age (year)	23 ± 1	23 ± 2	23 ± 2
Height (cm)	175 ± 5	163 ± 4	169 ± 8
Weight (kg)	69 ± 8	53 ± 6	61 ± 11
Body mass index (kg/m^2^)	22 ± 3	20 ± 2	21 ± 3
Heart rate (beats/min)	77 ± 11	78 ± 11	78 ± 11
Systolic blood pressure (mmHg)	123 ± 10	107 ± 7	116 ± 12
Diastolic blood pressure (mmHg)	76 ± 8	70 ± 6	73 ± 8

Note: data are expressed as mean +/− SD.
